# The Transcription Factor TCF1 Preserves the Effector Function of Exhausted CD8 T Cells During Chronic Viral Infection

**DOI:** 10.3389/fimmu.2019.00169

**Published:** 2019-02-12

**Authors:** Yifei Wang, Jianjun Hu, Yiding Li, Minglu Xiao, Haoqiang Wang, Qin Tian, Zhirong Li, Jianfang Tang, Li Hu, Yan Tan, Xinyuan Zhou, Ran He, Yuzhang Wu, Lilin Ye, Zhinan Yin, Qizhao Huang, Lifan Xu

**Affiliations:** ^1^The First Affiliated Hospital, Biomedical Translational Research Institute and School of Pharmacy, Jinan University, Guangzhou, China; ^2^Institute of Immunology, Third Military Medical University, Chongqing, China; ^3^Chengdu Military General Hospital, Chengdu, China; ^4^Department of Immunology, School of Basic Medicine, Tongji Medical College, Huazhong University of Science and Technology, Wuhan, China; ^5^Guangxi Key Laboratory of Biological Targeting Diagnosis and Therapy Research, National Center for International Research of Biological Targeting Diagnosis and Therapy, Guangxi Medical University, Nanning, China

**Keywords:** TCF1, CD8 T cell, exhaustion, LCMV, chronic viral infection

## Abstract

The long-term persistence of viral antigens drives virus-specific CD8 T cell exhaustion during chronic viral infection. Yet exhausted, CD8 T cells are still endowed with certain levels of effector function, by which they can keep viral replication in check in chronic infection. However, the regulatory factors involved in regulating the effector function of exhausted CD8 T cell are largely unknown. Using mouse model of chronic LCMV infection, we found that the deletion of transcription factor TCF-1 in LCMV-specific exhausted CD8 T cells led to the profound reduction in cytokine production and degranulation. Conversely, ectopic expression of TCF-1 or using agonist to activate TCF-1 activities promotes the effector function of exhausted CD8 T cells. Mechanistically, TCF-1 fuels the functionalities of exhausted CD8 T cells by promoting the expression of an array of key effector function-associated transcription regulators, including Foxo1, Zeb2, Id3, and Eomes. These results collectively indicate that targeting TCF-1 mediated transcriptional pathway may represent a promising immunotherapy strategy against chronic viral infections by reinvigorating the effector function of exhausted virus-specific CD8 T cells.

## Introduction

The CD8 T cell immune response is an important component of the adaptive immunesystem. Upon pathogen invasion, antigen-presenting cells, such as dendritic cells (DCs), will process pathogens, and present antigen peptides to T cells in an MHC-restricted manner. The binding of the peptide-MHC complex to TCR, together with costimulatory molecules and cytokines, provides stimulating signals to T cells, which are vital for T cell activation. After activation, naïve CD8 T cells differentiate into functional cytotoxic T lymphocytes (CTLs, effector CD8 T cells) within secondary lymphoid tissue (1). Then, effector CD8 T cells recognize class I MHC-peptide (pMHC I) complexes on specific target cells in the periphery and ultimately induce the destruction of target cells.During acute viral infection, the majority of effector CD8 T cells die by means of apoptosis after antigen clearance; only ~5–10% of the cells survive and differentiate into memory CD8 T cells to establish long-term immune protection (2). On the other hand, viruses, such as HIV (human immunodeficiency virus), HBV (Hepatitis B virus), and HCV (Hepatitis C virus), evolved multiple strategies to escape from the surveillance of the immune system (3). As a result, those pathogens cannot be cleared effectively and achieve a high viral load in the host, placing CD8 T cells under sustained antigen exposure, and eventually leading to CD8 T cell exhaustion.

T cell exhaustion is a state of antigen-specific T cell dysfunction and subsequent physical deletion (4–6). During exhaustion, CD8 T cells upregulate an array of inhibitory receptors, including PD-1, LAG3, Tim3, CD160, and 2B4, and progressively lose effector functions and proliferative capacity. The loss of effector functions during CD8 T cell exhaustion occurs in a hierarchical manner: IL-2 production, high proliferation, and cytotoxic capacity are lost first, then the ability to produce tumor necrosis factor is often lost at an intermediate stage of exhaustion; advanced exhaustion leads to the partial or complete loss of the ability to produce large amounts of interferon-gamma (IFN-γ) or beta-chemokines or the ability to degranulate, which can be indicated by CD107a/b expression, and the final stage of exhaustion is the physical deletion of viral-specific T cells (6). However, exhausted CD8 T cells are not inert. Previous studies demonstrated that exhausted CD8 T cells retain suboptimal but crucial functions that limit viral replication in both SIV (Simian Immunodeficiency Virus) animal models and HIV patients (7–9) and even reserve certain function of restricting cancer progress (10). The underlying mechanisms of CD8 T cell exhaustion remain largely unclear. Recently, several transcription factors, such as T-bet, Eomes, FOXO1, NFAT, and Blimp (11–15), as well as cell metabolism (16, 17), and epigenetic (18, 19) regulation, have been reported to be involved in the regulation of CD8 T cell exhaustion. Regardless, the mechanisms by which exhausted CD8 T cells retain a certain level of function to restrict pathogens remain unclear.

The transcription factor T cell factor 1 (TCF1), encoded by *Tcf7*, is the downstream transcription factor of the canonical Wnt signaling pathway. TCF1 is important for T cell development and maturation (20). For CD4 T cell subsets, TCF1 has been reported to promote cell lineage commitment to the T helper type 2 (Th2) fate; however, TCF1 inhibits T helper type 1 (Th1) differentiation by inducing the expression of the transcription factor GATA3 and subsequently IL-4 while repressing interferon-γ (IFN-γ) production (21). We previously found that TCF1 functions as an important hub upstream of the Bcl6-Blimp1 axis to initiate and secure the differentiation of follicular helper T cells (Tfh) (22). For CD8 T cells, TCF1 favors the formation of memory CD8 T cells in a listeria infection model via inducing the expression of the transcription factor Eomes through direct binding (23). Recently, several groups have confirmed that during chronic viral infection, CD8 T cells expressing high levels of TCF1 (TCF1^high^) exhibited a stem-cell-like phenotype, which maintained a better proliferative capacity and could further differentiate into both TCF1^high^ and TCF1^low^ CD8 T cell subsets (24–26). Based on previous reports, we speculate that TCF1 may also play an important role in the regulation of CD8 T cell function during chronic infection.

In the present study, we investigated the role of TCF1 in regulating the effector function of exhausted CD8 T cells during LCMV chronic infection. We found that a substantial group of viral-specific CD8 T cells continually expressed TCF1 during chronic viral infection. The expression level of TCF1 positively correlated with the cytokine-producing ability of CD8 T cells and negatively correlated with the expression of inhibitory receptors of CD8 T cells. Using *Tcf7* knock-out mice and bone marrow chimeras, we demonstrated that a TCF1 deficiency in CD8 T cells intrinsically resulted in a decreased cell number and impaired the cytokine-producing capacity of antigen-specific CD8 T cells during LCMV chronic infection. A distinct transcriptional signature in TCF1-deficient CD8 T cells compared to WT CD8 T cells during chronic infection, indicating that TCF1 maintains the exhausted CD8 T cell transcriptional programming. The upregulation of TCF1 expression substantially increased the number of viral-specific CD8 T cells and enhanced their cytokine-producing ability. In summary, we found that TCF1 plays an important role in the maintenance of the viral-specific CD8 T cell pool as well as their effector function during chronic viral infection. We speculate that TCF1 can be exploited as a potential therapeutic target, through which we may be able to optimize the T cell immune response during chronic viral infections, such as HIV and even tumorigenesis.

## Materials and Methods

### Mice, Virus, and GK1.5/Tamoxifen Treatment

*Tcf7*^*fl*/*fl*^ mice were provided by H.H. Xue (University of Iowa) with permission from the Institute Clinique de la Souris (part of the International Knockout Mouse Consortium). P14 (CD45.1) mice were provided by R. Ahmed (Emory University). Mice with transgenic expression of *Cd4-Cre*, mice with the ERT2-Cre knock-in, and C57BL/6J (CD45.1 and CD45.2) mice were purchased from Jackson Laboratory. The lymphocytic choriomeningitis virus (LCMV) clone 13 strain (Cl13) was provided by R. Ahmed (Emory University), and 2 × 10^6^ plaque-forming units (PFU) of virus were used to establish chronic infection in mice via intravenous injection. Wild-type mice, *Tcf* ^*fl*/*fl*^-CD4Cre mice, and their control littermates were infected at 6–10 weeks of age. Bone marrow chimeras were infected after 8–10 weeks of reconstitution. Mice infected with LCMV were housed in accordance with the institutional biosafety regulations of the Third Military Medical University. The anti-mouse CD4 antibody (GK1.5; BioXcell) was used to deplete CD4 T cells in mice via intravenous injection at a dose of 500 μg per mouse on day −1 and day 1 after LCMV Cl13 infection. Tamoxifen (T5648; Sigma-Aldrich) was injected intraperitoneally into mice at a dose of 1 mg per day per mouse. All mouse experiments were conducted in accordance with the guidelines of the Institutional Animal Care and Use Committees of the Third Military Medical University. This study was specifically reviewed and approved by the Laboratory Animal Welfare and Ethics Committee of the Third Military Medical University.

### Flow Cytometry and Antibodies

Flow cytometry data were acquired with a FACSCanto II (BD Biosciences) and were analyzed with FlowJo software (Tree Star). The antibodies and reagents used for flow cytometry staining are listed in Supplementary Table 1. For the detection of cytokine production, lymphocytes were stimulated for 5 h in the presence of the indicated peptide (0.2 μg/ml), Golgi Plug, Golgi Stop, anti-CD107a, and anti-CD107b antibodies (BD Biosciences). Intracellular cytokine staining for CD107, granzyme B, and Ki67 were performed with the Cytofix/Cytoperm Fixation/Permeabilization Kit (554714, BD Biosciences). Staining of TCF1, Id2, c-Myc, Nurr77, T-bet, Bcl6, EZH2, Eomes, and Bilmp1 was performed with the Foxp3/Transcription Factor Staining Buffer Set (00-5523; eBioscience). Staining for surface markers was performed in FACS buffer [PBS containing 2% BSA or FBS (w/v)]. Major histocompatibility complex (MHC) class I (H-2D^b^) tetramer specific for LCMV GP33-41 was provided by the tetramer core facility of the US National Institutes of Health (Emory). The MHC class I tetramer staining was performed by incubating the tetramer with cells for 30 min on ice.

### Virus Titration

The LCMV Cl13 viral loads in the tissue samples were quantified via quantitative RT-PCR as previously described (27). After weighing and homogenizing harvested tissues from infected mice, total RNA was extracted from the tissue homogenate using an RNeasy Micro Kit (QIAGEN) and was subjected to reverse transcription using the ReverAid Minus First Strand cDNA Synthesis Kit (Thermo) following the manufacturer's instructions. A LCMV-specific glycoprotein primer (GP-R: 5′-GCAACTGCTGTGTTCCCGAAAC-3′) was used for cDNA synthesis. The qPCR assay with LCMV glycoprotein-specific primer pairs (GP-R, 5′-GCAACTGCT GTGTTCCCGAAAC-3′, and GP-F 5′-CATTCACCTGGAC TTTGTCAGACTC-3′) was used to evaluate the viral loads in the tissue samples. The Cq (quantification cycle) values from RNA samples of 10-fold serially diluted LCMV-Armstrong virus that was already titrated by plaque assay was used to establish a standard curve. The pfu of LCMV-Cl13 in tissues was calculated with the following formula: lg(pfu) = slope^*^Cq+y-intercept; pfu/g = pfu calculated from above/tissue weight.

### BrdU Incorporation Assay

Infected mice were injected with a BrdU working solution at a dosage of 100 mg/kg body weight via intraperitoneal route. Mice were sacrificed approximately 3 h after BrdU injection. Then, the spleen was removed to isolate lymphocytes and conduct a flow cytometry analysis using a BrdU-APC Kit (BD Biosciences) following the manufacturer's instructions. Samples were acquired with a FACSCanto II (BD Biosciences) and were analyzed with FlowJo software (Tree Star). The BrdU-positive population indicates the newly proliferating cell subset after BrdU incorporation.

### Retroviral Constructs and Transduction

The retroviral constructs MIGR1 (MSCV-IRES-GFP) and pMKO.1-GFP were obtained from H. H. Xue (University of Iowa) and R. Ahmed (Emory University), respectively. The *Tcf7* coding sequence (two isoforms, P33 and P45) was cloned into the backbone of MIGR1 to overexpress TCF1 in CD8 T cells. All sequences were verified by DNA sequencing. Retroviruses were packaged by transfection of 293T cells with the retroviral vectors and packaging plasmids pCL^eco^ and pMD2G. P14 cells were activated *in vivo* by the injection of 200 μg of peptide (LCMV glycoprotein amino acids 33–45) into P14 mice. Activated P14 cells were infected for 90 min at 37°C by centrifugation at 800 g with freshly harvested retrovirus supernatants, 8 μg/ml polybrene (H9268; Sigma-Aldrich) and 20 ng/ml IL-2 (130-098-221; Miltenyi Biotec). The transduced P14 cells were transferred into recipient mice, followed by infection of the host with LCMV Cl13.

### Adoptive Transfer and Generation of Bone Marrow Chimeras

A total of 2 × 10^3^ naïve CD45.1 P14 cells (or retrovirus-transduced P14 cells) was adoptively transferred into naïve wild-type (CD45.2) mice, which were infected intravenously with 2 × 10^6^ PFU of LCMV Cl13 strain on the following day. Bone marrow was collected from *Tcf7*^*fl*/*fl*^-CD4Cre or *Tcf7*^*fl*/*f*^ERT2-Cre mice (CD45.2) and C57BL/6J wild-type mice (CD45.1). Approximately 5 × 10^6^ cells of a 4:6 mixture of *Tcf7*^*fl*/*fl*^CD4Cre or *Tcf7*^*fl*/*f*^ERT2-Cre bone marrow and WT bone marrow were transferred intravenously into lethally irradiated (two doses of 550 rads each) WT recipients. Recipient mice were reconstituted for 8–10 weeks before infection with LCMV.

### Quantitative RT-PCR

For comparison of gene expression in *Tcf7*^−/−^ CD8 T cells and wild-type CD8 T cells, antigen-experienced exhausted CD8 T cells (CD44^hi^PD1^hi^) at 15 days after infection were sorted and subsequently lysed in TRIzol LS reagent (10296; Life Technologies). Total RNA was extracted and reverse-transcribed with a RevertAid H Minus First Strand cDNA Synthesis Kit (K1632; Thermo Scientific). The resulting cDNA was analyzed for the expression of various target genes with a QuantiTect SYBR Green PCR Kit (204143; Qiagen) and the corresponding primers on a CFX96 Touch Real-Time System (Bio-Rad) (Supplementary Table 2).

### Statistical Analysis

Statistical analysis was conducted with Prism 6.0 software (GraphPad). Heat map was generated via Morpheus (Morpheus, https://software.broadinstitute.org/morpheus). An unpaired two-tailed *t*-test with 95% confidence interval was used to calculate *P*-values. For retroviral transduction and bone marrow chimera experiments, a paired two-tailed *t*-test with 95% confidence interval was used to calculate *P*-values.

## Results

### TCF1 Is Continually Expressed in CD8 T Cells and Is Positively Correlated With Its Effector Functions During Chronic Viral Infection

**Figure 1:**
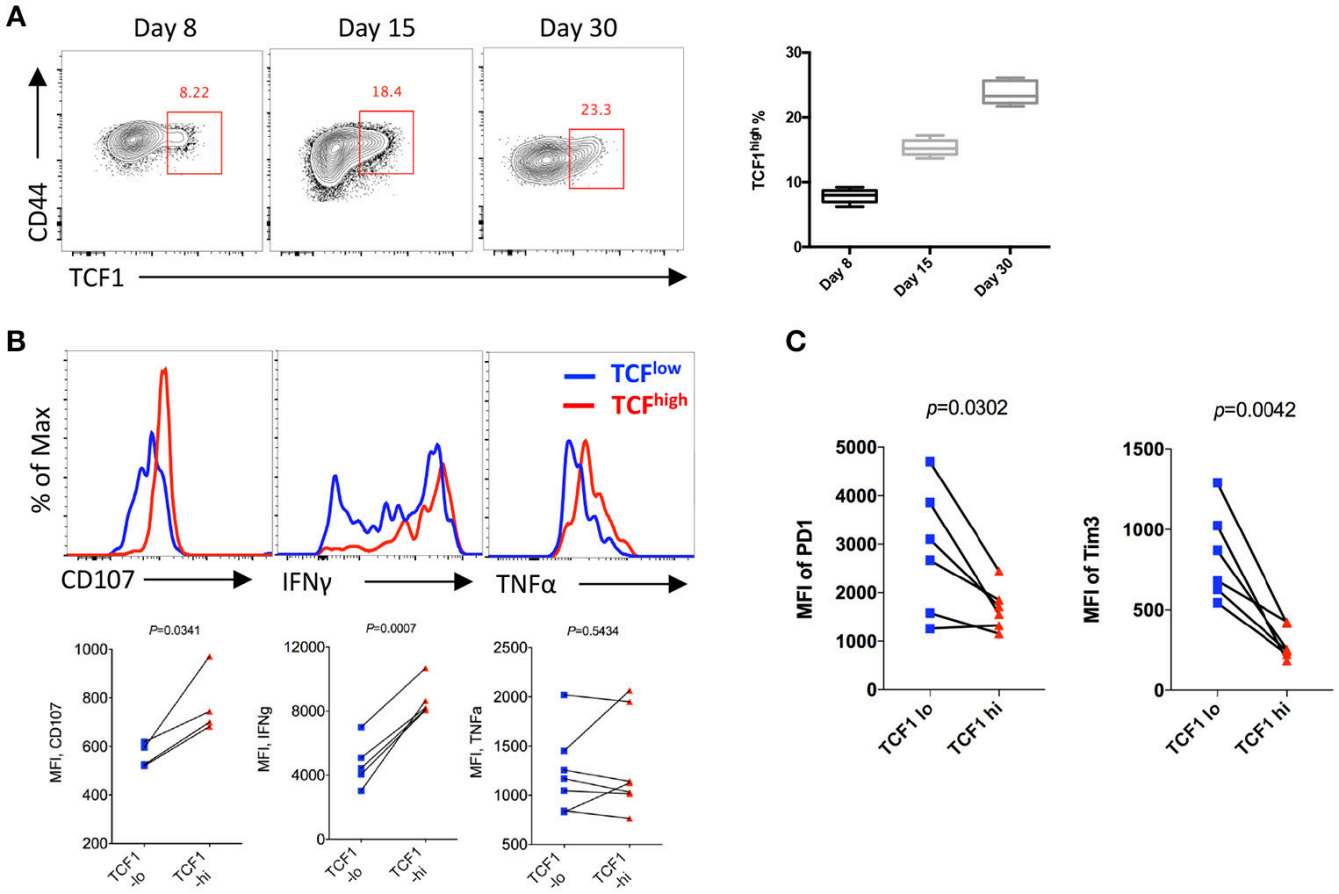
TCF1 is consistently expressed in viral specific CD8 T cell and correlated with CTL function. **(A)** Flow cytometry of viral specific CD8 T cells in the spleen of wild-type (CD45.2^+^) mice given adoptive transfer of P14 cells (CD45.1^+^), analyzed at either day 8, day 15, or day 30 after LCMV Cl13 infection. Percentages of TCF1^high^ P14 cells on each day post infection. **(B)** Flow cytometric histogram of CD107, IFNγ, or TNFα expression in TCF1^high^ (red) and TCF1^low^ (blue) P14 cells from the spleen of mice as in A at day 15 after LCMV Cl13 infection. **(C)** Quantification of the MFI of PD1 and Tim3 expression in TCF1^high^ (TCF1-hi) and TCF1^low^ (TCF1-lo) P14 cells from the spleen of mice as in A at day 15 after LCMV Cl13 infection. The *p*-value was calculated by a paired **(B,C)** *t*-test. Data are presentative of three independent experiments with at least four or five mice per group [error bar **(A)**, s.e.m.].

To analyze the kinetics of the expression of TCF1 in CD8 T cells and its correlation with CD8 T cell functions during LCMV chronic infection, P14 cells (CD45.1) expressing a GP33-specific transgenic T cell receptor were adoptively transferred into CD45.2 congenic wild-type (WT) C57BL/6J mice, then recipients were infected with LCMV Cl13 at 1 day after transfer. The results showed a sustained increase in the proportion of the TCF1^high^ population of P14 cells over time during chronic infection, and the percentage of TCF1^high^ P14 cells reached approximately 20% at the advanced stage (D30) of LCMV chronic infection (Figure 1A). Furthermore, we found that the expression levels of CD107a/b and IFNγ in transferred P14 cells were positively correlated with TCF1 expression at day 15 post infection with Cl13 (Figure 1B). The expression of inhibitory receptors, evidenced by PD1 and Tim3, in exhausted P14 cells was significantly higher in the TCF1^low^ population than that in the TCF1^high^ population (Figure 1C). These data suggested that a substantial group of viral-specific CD8 T cells continually express a high level of TCF1 during LCMV chronic infection, and TCF1 may serve as a positive regulator of effector function in exhausted CD8 T cells.

### *Tcf7* Deficiency Exacerbates CD8 T Cell Exhaustion in LCMV Chronic Infection

**Figure 2:**
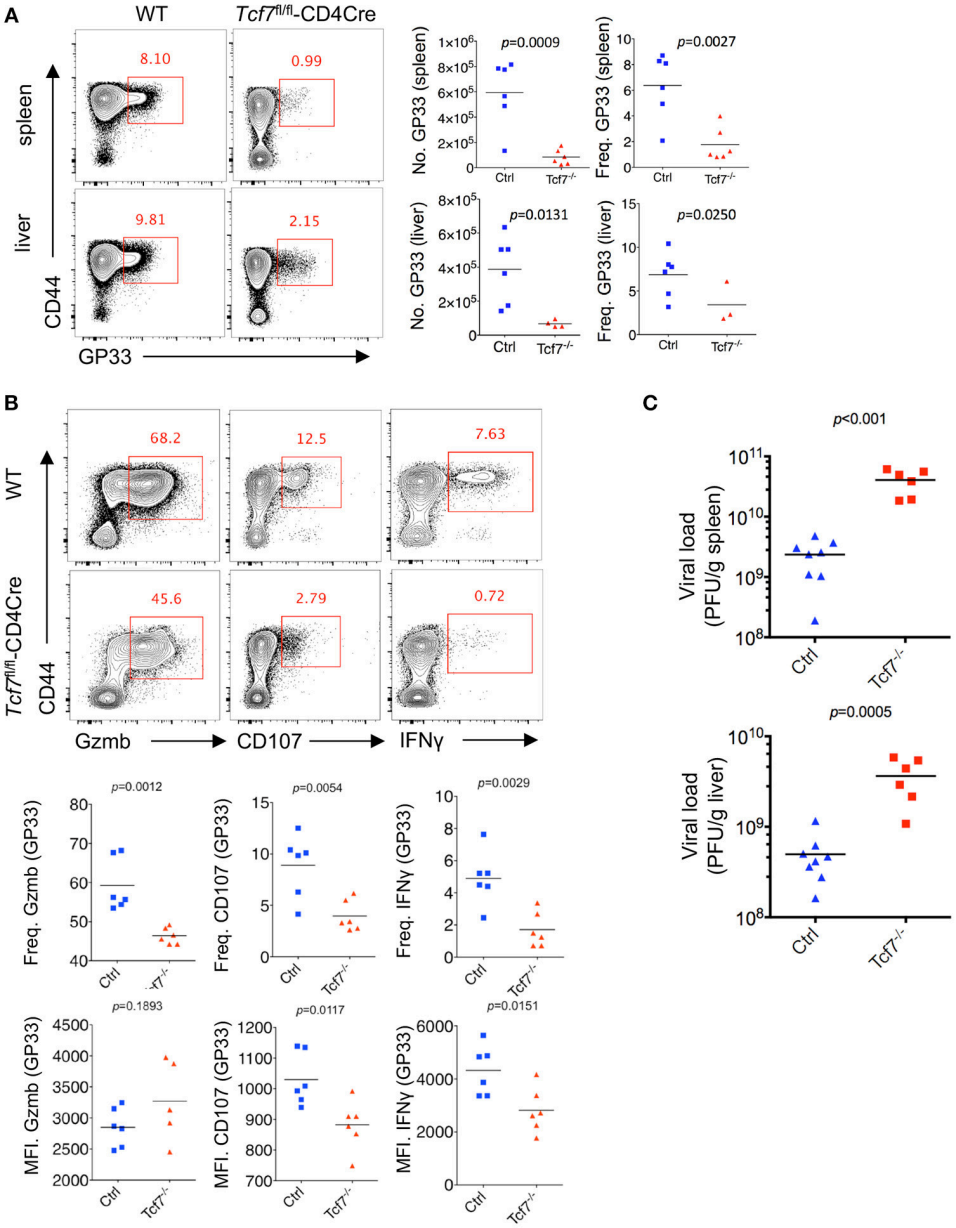
TCF1 deficiency exacerbates CD8 T cell exhaustion in LCMV chronic infection. **(A)** Flow cytometry of CD8 T cells in the spleen or liver of either wild-type (WT, Ctrl) mice or *Tcf7*^fl/fl^-CD4Cre mice (Tcf7^−/−^) at day 8 after LCMV Cl13 infection. Percentage of viral specific (GP33-tetramer positive) CD8 T cells was indicated in either WT (Ctrl) or Tcf7^−/−^ group. **(B)** Flow cytometry of CD8 T cells in the spleen or liver of either WT (Ctrl) mice or *Tcf7*^fl/fl^-CD4Cre mice (Tcf7^−/−^) at day 8 post LCMV Cl13 infection after stimulation with LCMV peptide GP33 *in vitro* for 5 h. Frequency of Gzmb-, CD107-, or IFNγ-positive CD8 T cells (up), and its summarized results (middle), MFI of Gzmb, CD107, or IFNγ was calculated in those positive cell population (down). **(C)** Summary of viral load in spleen and liver from either WT (Ctrl) mice or *Tcf7*^fl/fl^-CD4Cre (Tcf7^−/−^) mice at day 15 after LCMV Cl13 infection. The *p*-value was calculated by an unpaired *t*-test. Data are representative of three independent experiments with at least five mice per group.

Next, we crossed mice with *loxP*-flanked *Tcf7* alleles (*Tcf7*^fl/fl^) to mice expressing transgenic *Cre* recombinase from the T cell-specific *Cd4* promotor (CD4Cre) to generate mice with a conditional deletion of *Tcf7* in T cells (*Tcf7*^fl/fl^-CD4Cre mice) as we previously described (22). Both CD8 and CD4 T cells from *Tcf7*^fl/fl^-CD4Cre mice had largely abrogated TCF1 expression (Tcf7^−/−^ CD8 T cell). At day 8 post Cl13 infection, a significantly lower frequency and absolute number of either GP33- or NP396-tetramer-positive CD8 T cells, indicating viral-specific CD8 T cells, were observed in both the spleen and liver of *Tcf7*^fl/fl^-CD4Cre mice than those of WT (Tcf7^fl/fl^, WT) mice (Figure 2A, Supplementary Figure 1A).

We further conducted intracellular cytokine staining (ICS) of lymphocytes in the spleen of WT and Tcf7^−/−^ mice upon stimulation with a peptide (amino acids 33–41, GP33) of the LCMV glycoprotein *in vitro* at day 8 after Cl13 infection. A sharply decreased frequency of the Granzyme B-, CD107-, and IFNγ-positive population of CD8 T cells in *Tcf7*^fl/fl^-CD4Cre mice was observed compared to that in WT mice (Figure 2B). The Tcf7^−/−^ CD8 T cells also showed significantly decreased medium fluorescence intensity (MFI) of IFNγ and CD107 in the positive population. ICS was also conducted under the stimulation of two other LCMV antigen peptides, GP276, and NP396. A similar phenotype was observed, in which the absolute number and proportion of the Granzyme B-, CD107-, and IFNγ-positive population were dramatically lower in *Tcf7*^−/−^ CD8 T cells than those of the control group; the MFI of IFNγ and CD107 were similarly changed (Supplementary Figure 1B). In addition, we analyzed the viral load at day 15 post Cl13 infection in either *Tcf7*^fl/fl^-CD4Cre mice or WT mice after excluding the CD4 T cell influence via injection of the CD4 T cell-depleting antibody GK1.5 at 1 day before infection. Significantly higher viral titers of Cl13 in *Tcf7*^fl/fl^-CD4Cre mice than that in WT mice were observed (Figure 2C) which suggested that Tcf7-knock out in CD8 T cell further impaired the capacity to limit viral burden of exhausted CD8 T cells in a CD4 T cell-independent manner. The above data indicated that TCF1 positively regulates viral-specific CD8 T cell expansion and cytokine production during LCMV chronic infection, and the absence of TCF1 leads to the significant attenuation of viral-specific CD8 responses and a more exacerbated capacity to sustain viral replication during LCMV chronic infection.

### TCF1 Intrinsically Regulates Exhausted CD8 T Cell Proliferation, Survival, and Cytokine Production

To eliminate the influence of abnormal CD4 T cell responses attributed to *Tcf7* deficiency on CD8 T cell function during infection, we depleted CD4 T cells via injecting mice with the depleting antibody GK1.5 before LCMV infection (Supplementary Figure 2A). We noted that CD4 T cells were barely detected in mice after GK1.5 administration (Supplementary Figure 2B). Without CD4 T cells, a significant decrease in the frequency and total number of GP33-tetramer positive *Tcf7*^−/−^ CD8 T cells in the spleen and liver tissues was observed compared to that in WT counterparts at day 8 post Cl13 infection (Supplementary Figure 2C). Viral-specific CD8 T cells expressed substantially high levels of PD1; however, the Tim3 and PD1 double-positive population (PD1^+^Tim3^+^) of viral-specific CD8 T cells in *Tcf7*^fl/fl^-CD4Cre mice was significantly higher than that in WT mice (Supplementary Figure 2D). Upon LCMV-GP33 peptide stimulation *in vitro*, the frequency of Granzyme B-, CD107-, and IFNγ-positive CD8 T cells sharply decreased in *Tcf7*^−/−^ CD8 T cells compared with that in WT CD8 T cells (Supplementary Figure 2E).

**Figure 3:**
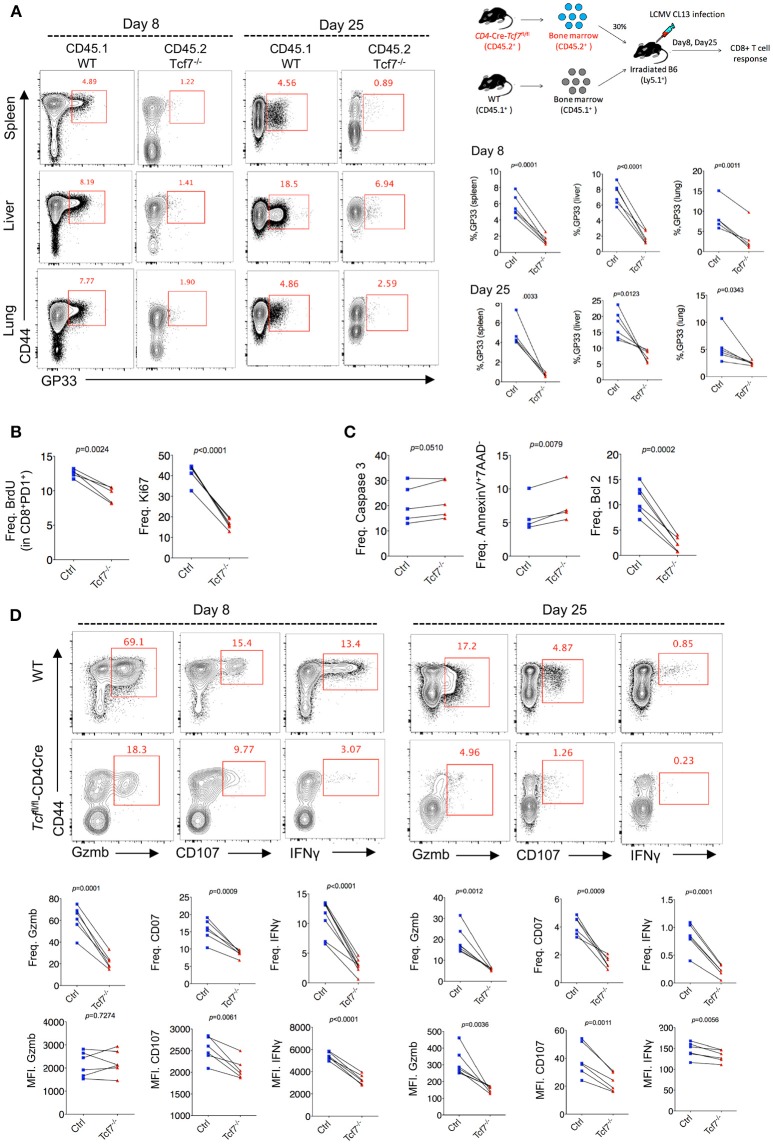
TCF1 intrinsically regulates CD8 T cell responses in LCMV chronic infection. **(A)** Experimental setup of establishment of bone marrow chimera mice via reconstituting irradiated WT recipient mice (CD45.1) with a mixture of congenitally marked bone marrow cells from *Tcf7*^fl/fl^-CD4Cre donor mice (CD45.2, 30%) and WT donor mice (CD45.1, 70%) (upper right). Percentage of viral specific (GP33-tetramer positive) CD8 T cells in either WT (CD45.1) or *Tcf7*^fl/fl^-CD4Cre (Tcf7^−/−^, CD45.2) cell population in the spleen, liver, or lung at day 8 or day 25 post LCMV Cl13 infection. **(B)** Summary of frequency of recently proliferating exhausted CD8 T cells via incorporating BrdU and Ki67 staining in PD1^+^ CD8^+^ T cells in the spleen of either WT (Ctrl) mice or *Tcf7*^fl/fl^-CD4Cre mice (Tcf7^−/−^) at day 25 after LCMV Cl13 infection. **(C)** Summary of frequency of Caspase 3-, Bcl2-postive, and apoptotic (Annexin V^+^7AAD^−^) CD8 T cells in the spleen of either WT (Ctrl) mice or *Tcf7*^fl/fl^-CD4Cre mice (Tcf7^−/−^) at day 25 after LCMV Cl13 infection. **(D)** Flow cytometry of CD8 T cells in the spleen or liver of either WT (Ctrl) mice or *Tcf7*^fl/fl^-CD4Cre mice (Tcf7^−/−^) at day 8 and day 25 post LCMV Cl13 infection after stimulation with LCMV peptide GP33 *in vitro* for 5 h. Percentage of Gzmb-, CD107-, or IFNγ-positive CD8 T cells (up), and summarized results (medium), MFI of Gzmb, CD107, or IFNγ was calculated in those positive cell population (down). The *p*-value was calculated by a paired *t*-test. Data are representative of three independent experiments with at least four or five mice per group.

To further confirm the intrinsic regulation of TCF1 on CD8 T cells during chronic viral infection, we generated bone marrow chimera mice by reconstituting irradiated WT recipient mice (CD45.1) with a mixture of congenitally marked bone marrow cells from *Tcf7*^fl/fl^-CD4Cre donor mice (CD45.2, 30%) and WT donor mice (CD45.1, 70%) (Figure 3A). GP33-tetramer staining data showed a dramatically decreased frequency of tetramer-positive viral-specific CD8 T cells in the Tcf7^−/−^ CD8 T cell (CD45.2) population than that in the WT (CD45.1) population at both day 8 and day 25 post Cl13 infection (Figure 3B), suggesting that TCF1 is intrinsically required for CD8 T cell expansion and survival during LCMV chronic infection. This phenotypic difference is consistent in CD8 T cells from spleen and nonlymphoid tissues, such as the liver and lung (Figure 3B). In addition, a BrdU incorporation assay at day 25 post infection with Cl13 further indicated that the proliferation of Tcf7^−/−^-exhausted CD8 T cells (PD1^+^CD8^+^) was significantly slower than that in the WT group (Figure 3B), which corresponds with the sharp decrease in the expression level of the cellular proliferation marker Ki67 in Tcf7^−/−^ CD8 T cells (Figure 3B). Meanwhile, despite the mild upregulation of Caspase 3, the frequency of apoptotic cells (AnnexinV^+^7AAD^−^) in Tcf7^−/−^ CD8 T cells was significantly higher than that in WT CD8 T cells (Figure 3C). The anti-apoptosis marker Bcl2 was dramatically decreased in Tcf7^−/−^ CD8 T cells compared to that in WT CD8 T cells (Figure 3C). Consistently, the ICS data presented substantial attenuation of Granzyme B, CD107, and IFNγ production in Tcf7^−/−^ CD8 T cells compared to WT CD8 T cells at both day 8 and day 25 after Cl13 infection (Figure 3D). The above data suggested that TCF1 is intrinsically crucial for maintaining the cell population and producing cytokines in exhausted CD8 T cells.

### TCF1 Regulates CD8 T Cell Function at Both Early and Advanced Stages

**Figure 4:**
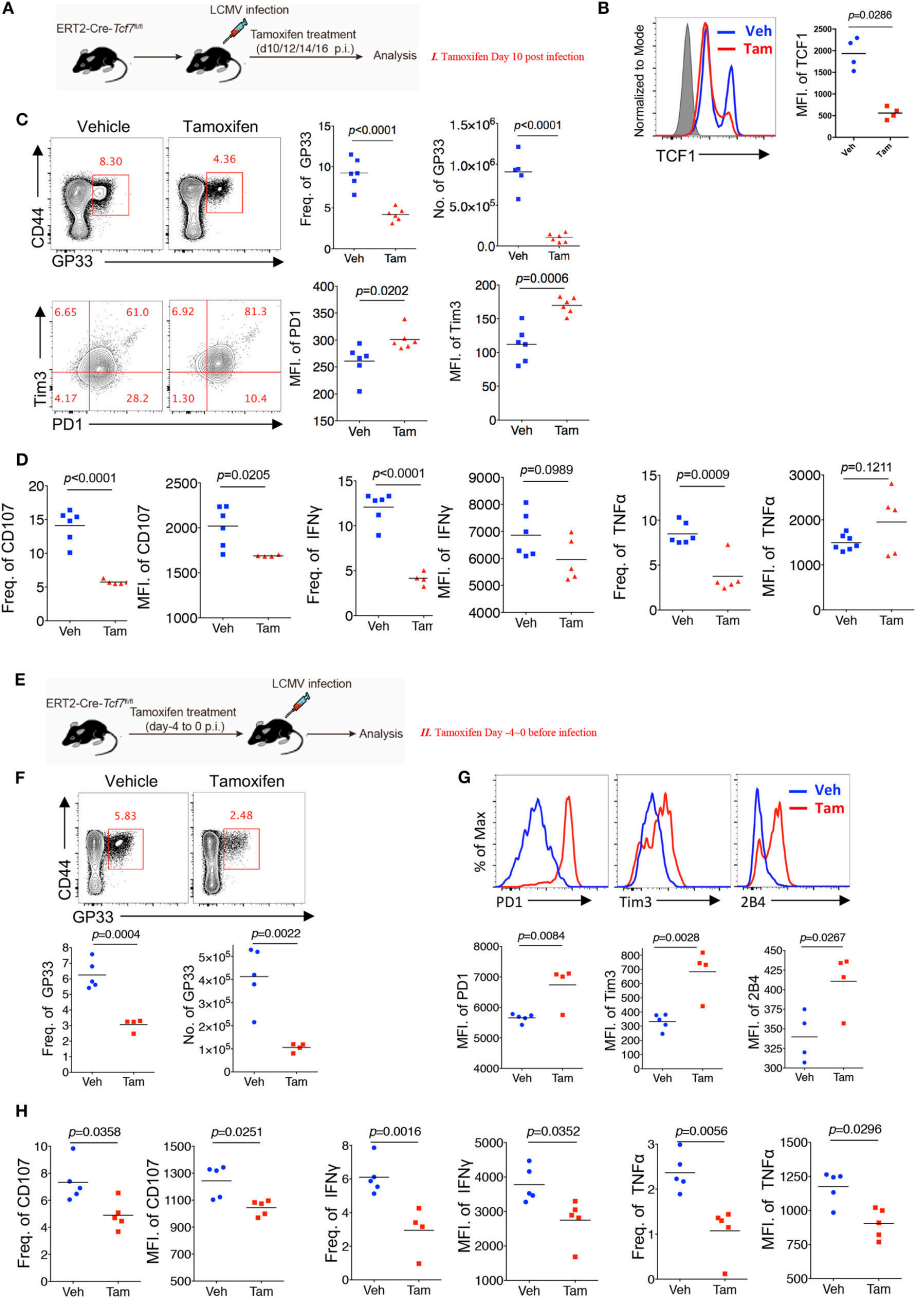
TCF1 regulates CD8 T cell responses at both early and advanced stage in LCMV chronic infection. **(A)** Experimental setup of inducible knock-out of TCF1 in *Tcf7*^fl/fl^-ERT2Cre mice at day 10~16 post LCMV Cl13 infection [strategy I **(C,D)**, delete *Tcf7* at advanced stage of chronic infection] via intraperitoneal injection with tamoxifen. **(B)** Flow cytometric analysis of TCF1 expression in CD8 T cell of tamoxifen-treated WT (Ctrl) mice or *Tcf7*^fl/fl^-ERT2Cre (Tcf7^−/−^) mice at day 25 after LCMV Cl13 infection. **(C)** Flow cytometry of GP33-tetramer positive CD8 T cells (upper left) and expression of PD1 and Tim3 in these positive populations (low left) in the spleen of *Tcf7*^fl/fl^-ERT2Cre mice treated with either tamoxifen (Tam) or vehicle (Veh) alone (strategy I) at day 25 post LCMV Cl13 infection. **(D)** Frequency of CD107-, IFNγ-, or TNFα-positive cells in CD8 T cells and MFI of CD107, IFNγ TNFα in those positive populations in the spleen of mice as in **(C)**. **(E)** Experimental setup of inducible knock-out of TCF1 in *Tcf7*^fl/fl^-ERT2Cre mice at day-4~0 before infection with LCMV Cl13 strain [strategy II **(F–H)**, delete *Tcf7* at early stage of chronic infection] via intraperitoneal injection with tamoxifen. **(F)** Flow cytometry of CD8 T cells in the spleen of *Tcf7*^fl/fl^-ERT2Cre mice treated with either tamoxifen or vehicle alone (strategy II) at day 25 post LCMV Cl13 infection, frequency, and number of GP33-tetramer positive cells were indicated. **(G)** Flow cytometric histogram of PD1, Tim3, and 2B4 expression in GP33-tetramer positive CD8 T cells in the spleen of mice as in **(E,H)** CD107, IFNγ, and TNFα expression in GP33-tetramer positive CD8 T cells in the spleen of mice as in **(E)**. The *p*-value was calculated by an unpaired *t*-test. Data are representative of three independent experiments with at least five mice per group.

Chronic viral infection is a dynamic process balancing the metastable equilibrium between CD8 T cells and pathogens (3). Although several previous studies have demonstrated the memory- or progenitor-like function of TCF1^high^ CD8 T cells at the late stage of LCMV chronic infection (25, 26), whether TCF1 regulates viral-specific CD8 T cell responses at the early effector stage remains unclear. Based on the above results demonstrating the positive role of TCF1 in regulating CD8 T cell effector function, we further investigated the role of TCF1 in regulating CD8 T cell function during the effective stage via an inducible knock-out of *Tcf7*. We crossed mice expressing a tamoxifen-sensitive estrogen receptor variant fused to transgenic *Cre* recombinase (ERT2Cre) with *Tcf7*^fl/fl^ mice to generate *Tcf7*^fl/fl^-ERT2Cre mice for an inducible deletion of *Tcf7* at different phases of infection. Mice were intraperitoneally injected with tamoxifen at 10 days after Cl13 infection (Strategy I) or 4 days before Cl13 infection (Strategy II) to knock out TCF1 expression in T cells of *Tcf7*^fl/fl^ERT2-Cre mice at either the advanced or early stage of Cl13 chronic infection (Figures 4A,E). Flow cytometry data confirmed that TCF1 expression was efficiently reduced after tamoxifen treatment in *Tcf7*^fl/fl^-ERT2Cre CD8 T cells (Figure 4B).

When deleting *Tcf7* at an advanced stage of Cl13 chronic infection via intraperitoneally injecting mice with tamoxifen at 10~13 days after infection, we observed that the frequency and absolute number of the GP33-tetramer-positive CD8 T cell population were significantly decreased, and the expression levels of inhibitory receptors (PD1 and Tim3) on viral-specific CD8 T cells were significantly increased in *Tcf7*^fl/fl^-ERT2Cre mice at 25 days after infection (Figure 4C). Meanwhile, compared with the control *Tcf7*^fl/fl^-ERT2Cre mice that were treated with vehicle, the frequency of CD107-, IFNγ-, and TNFα-positive CD8 T cells from tamoxifen-treated *Tcf7*^fl/fl^ERT2-Cre CD8 T cells was significantly lower (Figure 4D, Supplementary Figure 3A). Furthermore, the MFI of CD107 in the positive populations dramatically decreased in tamoxifen-induced Tcf7^−/−^ CD8 T cells (Figure 4D). On the other hand, when deleting *Tcf7* at an early stage of Cl13 chronic infection (4 days before infection) (Figure 4E), a significantly decreased frequency and total number of GP33-tetramer-positive CD8 T cells in the tamoxifen-treated group were observed at 25 days after infection (Figure 4F), accompanying the prominent increase in the expression of inhibitory receptors, such as PD1, Tim3, and 2B4 (Figure 4G). According to ICS, a substantial attenuation of CD107, IFNγ, and TNFα production in *Tcf7*^−/−^ CD8 T cells was observed upon GP33 peptide stimulation *in vitro* (Figure 4H, Supplementary Figure 3A). In addition, we confirmed the intrinsic role of TCF1 in the regulation of CD8 T cells in the *Tcf7*^fl/fl^-ERT2Cre system via a bone marrow chimera model, which was treated with tamoxifen before (Strategy II) or after (Strategy I) Cl13 infection as described above (Supplementary Figure 3B). In this *Tcf7*^fl/fl^-ERT2Cre chimeric model, we observed a dramatically decreased frequency of GP33-tetramer-positive cells in *Tcf7*^fl/fl^-ERT2Cre CD8 T cells compared to that derived from WT donor CD8 T cells in both strategies (Supplementary Figure 3C). The CD107, IFNγ, and TNFα expression level in TCF1-deficient CD8 T cells was significantly lower than that of WT CD8 T cells (Supplementary Figure 3D). The above data suggested that TCF1 positively regulates viral-specific CD8 T cell function at both the early and advanced phases of LCMV chronic infection.

### Exhausted CD8 T Cells With TCF1 Deficiency Present Distinct Transcriptional Signatures During Chronic Infection

**Figure 5:**
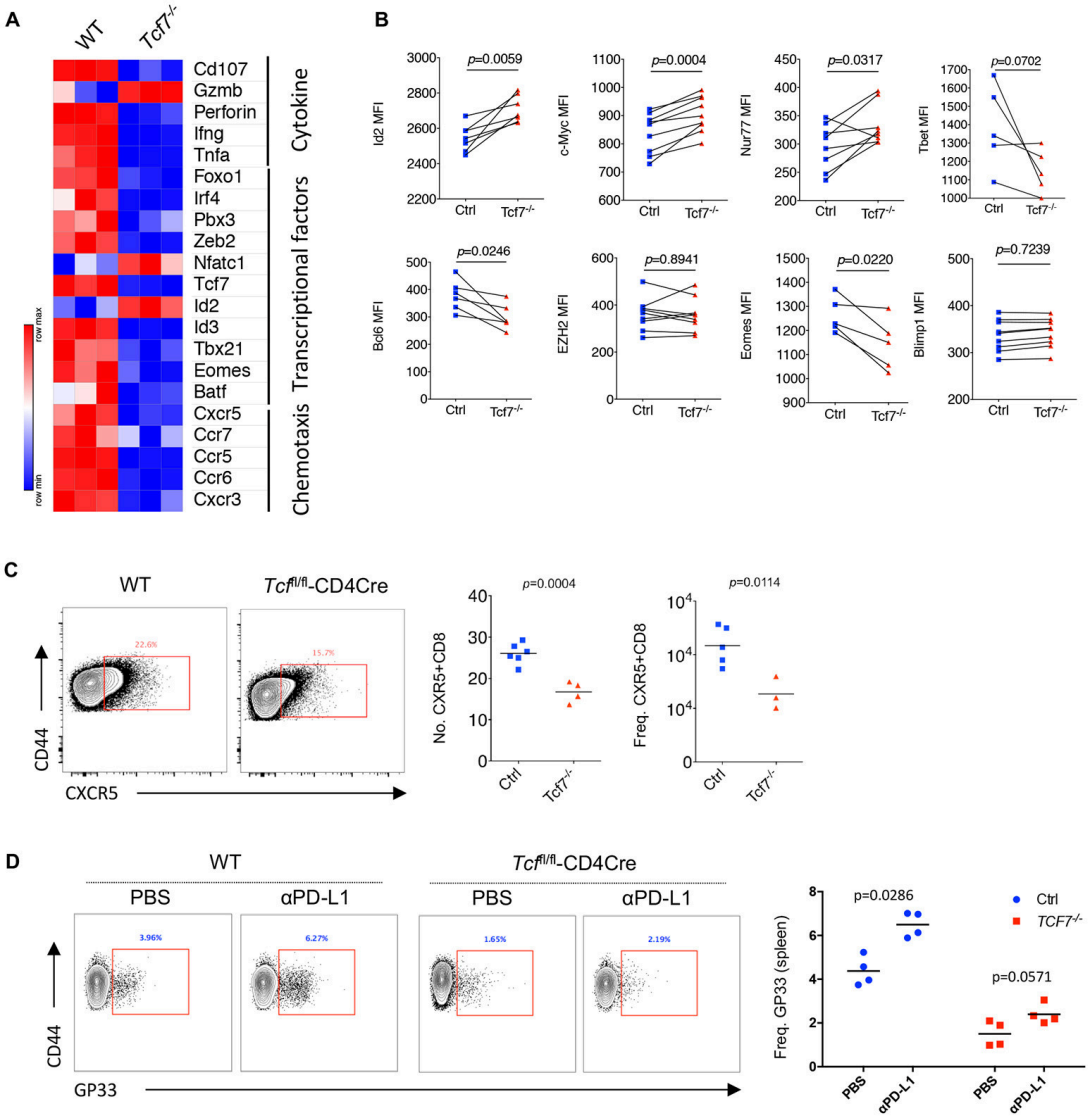
Distinct molecular signatures of TCF1 deficient CD8 T cells. **(A)** Heat map of selected genes expressed in exhausted WT and Tcf7^−/−^ CD8 T cells from mice infected with LCMV Cl13 strain. **(B)** Expression (calculated as MFI) of selected gene in viral specific CD8 T cells from spleen of bone marrow chimeras as in Figure 3A. **(C)** Number and frequency of CXCR5^+^CD8^+^ T cells in the spleen of either WT (Ctrl) mice or *Tcf7*^fl/fl^-CD4Cre mice (Tcf7^−/−^) at day 8 post LCMV Cl13 infection. **(D)** Frequency of GP33-tetramer positive CD8 T cells in the spleen of either WT (Ctrl) mice or *Tcf7*^fl/fl^-CD4Cre mice (Tcf7^−/−^) at day 15 post LCMV Cl13 infection following intravenous injection of αPD-L1 antibody at day 10 post-infection. The *p*-value was calculated by an unpaired *t*-test **(C,D)** or a paired *t*-test **(B)**. Data are representative of three independent experiments with at least three or four mice per group.

Previous studies demonstrated that exhausted CD8 T cells have unique gene expression profiles that are different from memory, naïve, or anergic CD8 T cells (28). Therefore, we sorted antigen-experienced exhausted CD8 T cells (CD44^hi^PD1^hi^) at 15 days after infection to extract total RNA of the different subsets of CD8 T cells and conduct array of quantitative real-time PCR (qRT-PCR) to profile the transcriptional signatures of exhausted *Tcf7*^−/−^ CD8 T cells. We divided the selected genes into three groups: genes in group A were related to cytokine production and cytolysis; genes in group B encode several transcription factors that were previously reported to be involved in the regulation of CD8 T cell responses to viral infection; and genes in group C were associated with CD8 T cell migration and chemotaxis. By qRT-PCR, we noted that the mRNA levels of cytokine and cytotoxic associated genes, such as *Cd107, Perforin, Ifn-*γ, *Tnfa*, were sharply reduced in exhausted *Tcf7*^−/−^ CD8 T cells than those in exhausted WT CD8 T cells (Figure 5A, Supplementary Figure 4A). The levels of transcription factors crucial for CD8 T cell differentiation and antiviral responses, such as *Foxo1, Zeb2, Id3*, and *Eomes*, also decreased (Figure 5A, Supplementary Figure 4A). Moreover, *Cxcr5*, which has been reported to positively associate with CD8 function during chronic infection (24, 29), was dramatically downregulated in exhausted *Tcf7*^−/−^ CD8 T cells (Figure 5A, Supplementary Figure 4A).

In the *Tcf7*^fl/fl^-CD4Cre bone marrow chimeras at 25 days after infection with LCMV Cl13 stain, we further confirmed the significantly reduced expression of Bcl6 and Eomes which have been confirmed to be crucial factors that regulate CD8 T cell functions (13, 26, 30–32) in exhausted *Tcf7*^−/−^ CD8 T cells via flow cytometry analysis (Figure 5B, Supplementary Figure 4B). In our previous study, we found that the CXCR5^+^ CD8 T cells with *Id2* deficiency express lower level of inhibitory receptors and produce higher effect factors (29). A significant decreased transcriptional level of *Id3* (Figure 5A) and increased expression level of Id2 (Figure 5B) detected via flow cytometry analysis were observed, suggesting that the imbalance of Id2/Id3 may involve in the deterioration of *Tcf7*^−/−^ exhausted CD8 T cells. The inconsistence of expression levels of mRNA (Figure 5A) and protein (Figures 2B, 3D, 5B) of some genes (e.g., *Gzmb, Id2, Ezh2*) may due to the different models and/or possible post-transcriptional regulation of those genes. In addition, the frequency and total number of CXCR5^+^ CD8 T cells, which were reported to play positive roles in chronic viral infection (24, 29), were significantly decreased in *Tcf7*^−/−^ CD8 T cells at 8 days of Cl13 chronic infection (Figure 5C). And consistently with previously researches, PD-1 pathway blockade via anti-PD-L1 antibody-treatment restored the population of exhausted viral-specific CD8 T cells (GP33-positive) in chronic infection (33) while *Tcf7*^−/−^ mice only presented a weak response to anti-PD-L1 antibody-treatment (Figure 5D). Above results indicated that TCF1 reprograms exhausted CD8 T cell transcriptional profiling and is crucial for CD8 T cell effector functions.

### Upregulation of TCF1 Enhanced CD8 T Cell Function During LCMV Chronic Infection

**Figure 6:**
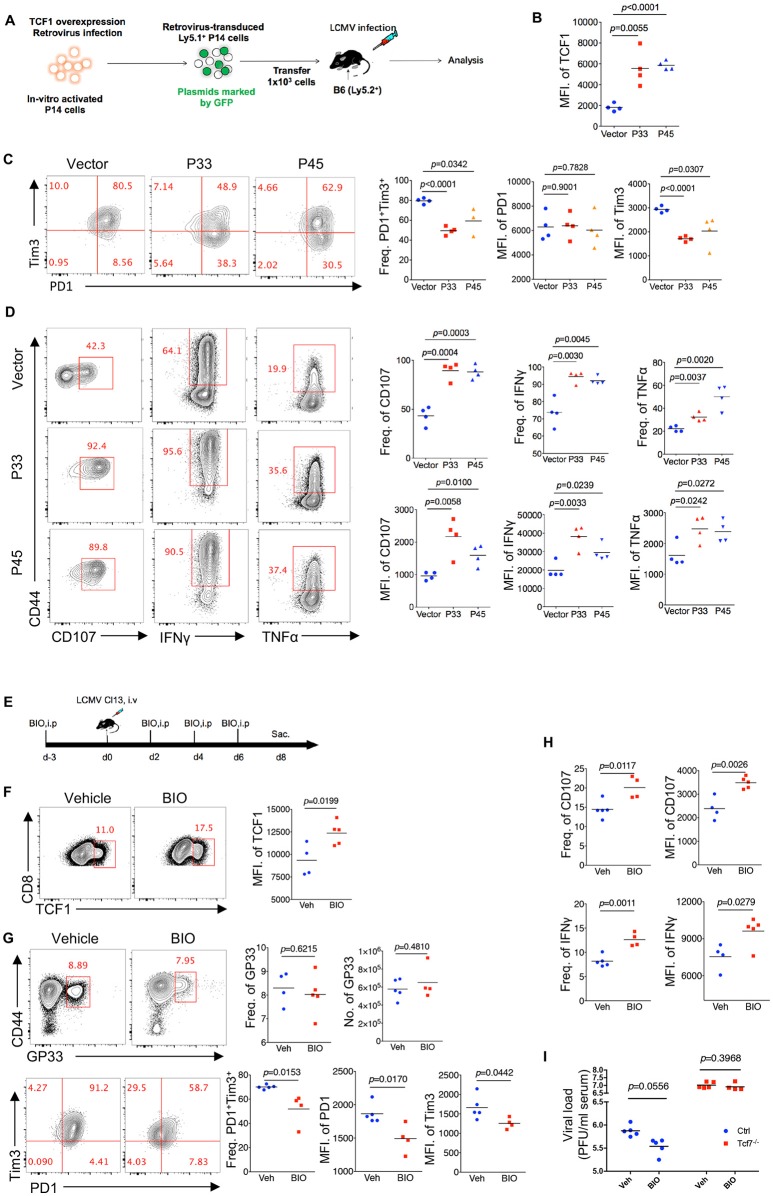
Upregulation of TCF1 enhanced exhausted CD8 T cell function. **(A)** Experimental setup of retroviruses mediated overexpressing P33 or P45 in viral specific CD8 T cells (P14 cells) and analysis of introduced P14 cell (GFP^+^) at day 8 post LCMV Cl13 infection. **(B)** MFI of TCF1 in GFP^+^ P14 cells in recipient mice at day 8 after LCMV Cl13 infection. **(C)** Flow cytometry of GFP^+^ P14 cells in the spleen of recipient mice as in **(B)** (left), and summary of the frequency of PD1+Tim3+ double positive cells and MFI of PD1 and Tim3 in those positive populations (right). **(D)** Flow cytometry of GFP^+^ P14 cells as in B, percentage of CD107-, IFNγ-, or TNFα-positive cells (left), and the summarized results (upper right). MFI of CD107, IFNγ, and TNFα were calculated in those positive populations (lower right). **(E)** Experimental setup of upregulation TCF1 expression via BIO treatment. **(F)** MFI of TCF1 in CD8 T cells in spleen of mice treated with BIO or vehicle at day 8 after LCMV Cl13 infection. **(G)** Flow cytometry of GP33-tetramer positive CD8 T cells (up) and expression of PD1 and Tim3 (down) in the spleen of mice treated with either BIO or vehicle (Veh) alone as in **(F)**. **(H)** Frequency of CD107- or IFNγ-positive cells in viral specific CD8 T cells and MFI of CD107, IFNγ in those positive populations in the spleen of mice as in **(F)**. **(I)** Summary of viral load in serum from either WT (Ctrl) mice or *Tcf7*^fl/fl^-CD4Cre (Tcf7^−/−^) mice at day 15 after LCMV Cl13 infection following intraperitoneal injection with either BIO or vehicle as **(E)**. The *p*-value was calculated by an unpaired *t*-test. Data are representative of three independent experiments with at least four mice per group.

Since TCF1 supports the CD8 T cell response against chronic viral infection in a cell-autonomous manner, in both the early phase (8 days post infection) and advanced phase (20 days post infection or later), by maintaining a virus-specific CD8 T cell pool as well as its effector function, we further investigated whether the upregulation of TCF1 could enhance the CD8 T cell response during LCMV chronic infection. A retroviral transduction system was used to overexpress the truncated (P33) and full-length (P45) isoforms of TCF1 in P14 cells (CD45.1). We transferred P14 cells, including transduced (GFP^+^) and nontransduced (GFP^−^) cells, into WT (CD45.2) recipient mice, and then infected recipient mice with Cl13 1 day after transfer (Figure 6A). We noted that at 8 days after Cl13 infection, the ectopic expression of either isoform of TCF1 was substantially enhanced in the transduced P14 cells (Figure 6B). We observed a significant decrease in the frequency of the PD1^+^Tim3^+^ population in transduced P14 cells than that in nontransduced P14 cells, and the MFI of Tim3 in its positive population agreed (Figure 6C). In addition, compared to the control group in which P14 cells were transduced with an empty retroviral vector, P14 cells overexpressing P33, or P45 substantially increased the frequency of the CD107-, IFNγ-, and TNFα-producing populations upon GP33 peptide stimulation *in vitro* (Figure 6D). The MFIs of CD107, IFNγ, and TNFα in cytokine-producing P14 cells were significantly enhanced by TCF1 overexpression as well (Figure 6D).

Since TCF1 is a downstream target of the canonical Wnt signaling pathway, we utilized the Wnt signaling agonist (2′Z,3′E)-6-Bromoindirubin-3-oxime (BIO) to activate the Wnt signaling pathway (23, 34) in mice infected with Cl13 (Figure 6E). The data showed that BIO administration sufficiently promotes the expression of TCF1 in CD8 T cells from treated mice (Figure 6F). At 8 days after Cl13 infection, a slightly increased number of GP33-tetramer-positive CD8 T cells and substantially decreased inhibitory receptor expression in BIO-treated mice were observed, compared to vehicle-treated control mice (Figure 6G). Significantly enhanced intracellular cytokine production in CD8 T cells from BIO-treated mice has also been observed in accordance with a TCF1 overexpression model (Figure 6H, Supplementary Figure 5). Furthermore, we found that BIO-treatment decreased viral load in Cl13-infected WT mice, but not in *Tcf7*^−/−^ counterparts (Figure 6I). The above data demonstrated that the upregulation of TCF1 expression is sufficient to alleviate CD8 T cell exhaustion via reducing inhibitor receptor (especially Tim3) expression and enhancing cytokine production in viral-specific CD8 T cells.

## Discussion

Although dysfunctional and hyposensitive, exhausted CD8 T cells still play an indispensable role in balancing persistent pathogen and host immune restriction (9). However, how exhausted CD8 T cells maintain their population and residual effector function remain unclear. In a previous study, we identified a population of CD8 T cells expressing CXCR5, which plays a critical role in controlling viral replication during LCMV Cl13 infection (29). This CXCR5-expressing subset was confirmed to contribute to the proliferation burst after PD1 blockade therapy in mice chronically infected with LCMV and that synchronously highly expressed TCF1 (24). Afterward, two groups suggested that the TCF1-expressing exhausted CD8 T cells are memory-like and sustain the long-term CD8 response to chronic viral infections via retaining the proliferative capacity, regeneration, and differentiation of CD8 T cells (25, 26). TCF1 is required for the differentiation and maintenance of the memory CD8 T cell pool (23, 35, 36). Except for the memory-like or progenitor-like roles of TCF1^high^ CD8 T cells during chronic viral infection, we wondered whether TCF1 regulates CD8 T cell effector function during chronic infection.

In the present study, the kinetics of TCF1 expression in viral-specific CD8 T cells during LCMV Cl13 chronic infection indicated that a population of exhausted CD8 T cells substantially and continually expresses TCF1 during chronic infection, which is consistent with previous studies suggesting that TCF1^high^ CD8 T cells exist in exhausted CD8 T cells and serve as progenitors to maintain the CD8 T cell population during chronic infection (25, 26). *Tcf7* knock-out in T lymphocytes results in attenuated cell proliferation, apoptosis tendency, and decreased production of both cytokine (IFNγ, TNFα) and degranulation-related markers (CD107a/b, granzyme B) in viral-specific CD8 T cells during chronic LCMV infection. Data from the inducible knock-out *Tcf7*^fl/fl^-ERT2Cre mice infected with Cl13 suggested that the positive role of TCF1 in the regulation of exhausted CD8 T cell effector functions occurred at both the early and advanced stages of chronic infection. Our study demonstrates that TCF1 supports sustaining exhausted CD8 T cell effector function, which is important for controlling viral replication during chronic viral infection. TCF1 is essential for T cell fate commitment and facilitates mature T cell responses (20, 37). TCF1 deficiency diminished effector CD8 T cell function only in modest ways during acute infection (23, 38); however, the roles of TCF1 in CD8 T cell effector function during chronic infection were usually overlooked despite the important role of exhausted viral CD8 T cells in limiting viral replication. Our present study demonstrated the importance of TCF1 during LCMV chronic infection as a positive regulator of CD8 T cell proliferation and cytokine production. We further analyzed the molecular signatures of TCF1 regulation and confirmed a distinct transcriptional landscape of exhausted TCF1-deficient CD8 T cells compared to exhausted WT CD8 T cells during LCMV chronic infection.

In both conventional (*Tcf7*^fl/fl^-CD4Cre) and inducible (*Tcf7*^fl/fl^-ERT2Cre) TCF1 knock-out models, the TCF1 deficiency significantly reduced the number of viral-specific CD8 T cells during LCMV chronic infection. The decreased expression level of the anti-apoptotic marker Bcl2 and the upregulation of a series of apoptosis-associated genes. A BrdU incorporation assay demonstrated that a TCF1 deficiency sharply inhibited exhausted CD8 T cell proliferation. The decreased frequency of cytokine-producing CD8 T cells in the TCF1-deficient group may be associated with the reduced population of viral-specific CD8 T cells to some extent. Nevertheless, the MFIs of cytokine (IFNγ, and TNFα) and degranulation markers (CD107, and sometimes Granzyme B) were significantly decreased in the positive population of Tcf7^−/−^ CD8 T cells, confirming that the TCF1 deficiency impaired the cytokine production capacity of viral-specific CD8 T cells during chronic viral infection. Based on previous reports, Id2, and Id3 interact with each other in balancing the effector and memory function of CD8 T cells during acute infection. In addition, the Blimp1-Id3-E2A axis plays a crucial role in regulating the terminal differentiation and memory establishment of T cells (39, 40). In the present study, we observed dramatically enhanced expression of Id2 and deceased transcriptional level of Id3 in Tcf7^−/−^ CD8 T cells compared to that in WT CD8 T cells. We speculate that TCF1 may regulate exhausted CD8 T cell effector function during chronic infection via influencing the equilibrium of Id2/Id3 (41), which needs to be further investigated to determine whether these hypothetical mechanisms function in regulating exhausted CD8 T cell responses during viral chronic infection. In addition, viral loads were significantly higher in mice infected with LCMV Cl13 and after ruling out CD4 T cell influence via GK1.5 depletion. The overexpression of TCF1 in viral-specific CD8 T cells, as well as upregulating TCF1 expression by BIO treatment, reduced the PD1^+^Tim3^+^ population in viral-specific CD8 T cells and significantly increased the expression of CD107, IFNγ, and TNFα. Taken together, these results indicated that TCF1 is crucial for CD8 T cells in limiting viral replication and conserving viral-specific CD8 T cell effector functions during LCMV chronic infection. Moreover, modulation of the TCF1 pathway may be a promising strategy to improve CD8 effector function during chronic viral infection.

In conclusion, we have established the curial role of TCF1 in the regulation of exhausted CD8 T cell effector function during chronic viral infection via retaining its proliferation capacity and cytokine-producing ability. These findings provide the novel possibility to reinvigorate exhausted CD8 T cell effector functions, which is important for controlling chronic infectious diseases and cancers.

## Author Contributions

LX, QH, ZY, YuW, and LY designed and oversaw experiments. QH, YiW, JH, YL, MX, HW, QT, ZL, JT, LH, YT, XZ, and RH performed experiments. QH and LX analyzed experiments. YiW, QH, and LY wrote the paper.

### Conflict of Interest Statement

The authors declare that the research was conducted in the absence of any commercial or financial relationships that could be construed as a potential conflict of interest.

## References

[1] Masopust D Schenkel JM. The integration of T cell migration, differentiation and function. Nat Rev Immunol. (2013) 13:309–20. 10.1038/nri3442 23598650

[2] Wherry EJ Teichgraber V Becker TC Masopust D Kaech SM Antia R. Lineage relationship and protective immunity of memory CD8 T cell subsets. Nat Immunol. (2003) 4:225–34. 10.1038/ni889 12563257

[3] Virgin HW Wherry EJ Ahmed R. Redefining chronic viral infection. Cell (2009) 138:30–50. 10.1016/j.cell.2009.06.036 19596234

[4] Gallimore A Glithero A Godkin AJ Tissot AC Pluckthun A Elliott T. Induction and exhaustion of lymphocytic choriomeningitis virus-specific cytotoxic T lymphocytes visualized using soluble tetrameric major histocompatibility complex class I-peptide complexes. J Exp Med. (1998) 187:1383–93. 10.1084/jem.187.9.1383 9565631 PMC2212278

[5] Zajac AJ Blattman JN Muralikrishna K Sourdive DJD Suresh M Altman JD. Viral immune evasion due to persistence of activated T cells without effector function. J Exp Med. (1998) 188:2205–13. 10.1084/jem.188.12.2205 9858507 PMC2212420

[6] Wherry EJ. T cell exhaustion. Nat Immunol. (2011) 12:492–9. 10.1038/ni.2035 21739672

[7] Price DA West SM Betts MR Ruff LE Brenchley JM Ambrozak DR. T cell receptor recognition motifs govern immune escape patterns in acute SIV infection. Immunity (2004) 21:793–803. 10.1016/j.immuni.2004.10.010 15589168

[8] Jones RB Walker BD. HIV-specific CD8+ T cells and HIV eradication. J Clin Invest. (2016) 126:455–63. 10.1172/JCI80566 26731469 PMC4731167

[9] Zehn D Utzschneider DT Thimme R. Immune-surveillance through exhausted effector T-cells. Curr Opin Virol. (2016) 16:49–54. 10.1016/j.coviro.2016.01.002 26826950

[10] Speiser DE Ho P Verdeil G. Regulatory circuits of T cell function in cancer. Nat Rev Immunol. (2016) 16:599–611. 10.1038/nri.2016.80 27526640

[11] Szabo SJ Kim ST Costa GL Zhang X Fathman CG Glimcher LH. A novel transcription factor, T-bet, directs Th1 lineage commitment. Cell (2000) 100:655–69. 10.1016/S0092-8674(00)80702-3 10761931

[12] Angelosanto JM Wherry EJ. Transcription factor regulation of CD8+ T-cell memory and exhaustion. Immunol Rev. (2010) 236:167–75. 10.1111/j.1600-065X.2010.00927.x 20636816

[13] Buggert M Tauriainen J Yamamoto T Frederiksen JW Ivarsson MA Michaelsson J. T-bet and eomes are differentially linked to the exhausted phenotype of CD8+ T cells in HIV infection. PLoS Pathog. (2014) 10:e1004251. 10.1371/journal.ppat.1004251 25032686 PMC4102564

[14] Staron M Gray SM Marshall HD Parish IA Chen JH Perry CJ. The transcription factor FoxO1 sustains expression of the inhibitory receptor PD-1 and survival of antiviral CD8 + T cells during chronic infection. Immunity (2014) 41:802–14. 10.1016/j.immuni.2014.10.013 25464856 PMC4270830

[15] Martinez GJ Pereira RM Aijo T Kim EY Marangoni F Pipkin ME. The transcription factor NFAT promotes exhaustion of activated CD8+ T cells. Immunity (2015) 42:265–78. 10.1016/j.immuni.2015.01.006 25680272 PMC4346317

[16] Chang C Pearce EL. Emerging concepts of T cell metabolism as a target of immunotherapy. Nat Immunol. (2016) 17:364–8. 10.1038/ni.3415 27002844 PMC4990080

[17] Scharping NE Menk AV Moreci RS Whetstone RD Dadey RE Watkins SC The tumor microenvironment represses T cell mitochondrial biogenesis to drive intratumoral T cell metabolic insufficiency and dysfunction. Immunity (2016) 45:374–88. 10.1016/j.immuni.2016.07.009 27496732 PMC5207350

[18] Pauken KE Sammons MA Odorizzi PM Manne S Godec J Khan O. Epigenetic stability of exhausted T cells limits durability of reinvigoration by PD-1 blockade. Science (2016) 354:1160–5. 10.1126/science.aaf2807 27789795 PMC5484795

[19] Sen DR Kaminski J Barnitz RA Kurachi M Gerdemann U Yates K. The epigenetic landscape of T cell exhaustion. Science (2016) 354:1165–9. 10.1126/science.aae0491 27789799 PMC5497589

[20] Yu Q Sharma A Sen JM. TCF1 and β-catenin regulate T cell development and function. Immunol Res. (2010) 47:45–55. 10.1007/s12026-009-8137-2 20082155 PMC2891409

[21] Maier E Hebenstreit D Posselt G Hammerl P Duschl A Horejshoeck J. Inhibition of suppressive T cell factor 1 (TCF-1) isoforms in naive CD4+ T cells is mediated by IL-4/STAT6 signaling. J Biol Chem. (2011) 286:919–28. 10.1074/jbc.M110.144949 20980261 PMC3019123

[22] Xu L Cao Y Xie Z Huang Q Bai Q Yang X The transcription factor TCF-1 initiates the differentiation of TFH cells during acute viral infection. Nat Immunol. (2015) 16:991–9. 10.1038/ni.3229 26214740

[23] Zhou X Yu S Zhao D Harty JT Badovinac VP Xue H. Differentiation and persistence of memory CD8+ T cells depend on T cell factor 1. Immunity (2010) 33:229–40. 10.1016/j.immuni.2010.08.002 20727791 PMC2928475

[24] Im SJ Hashimoto M Gerner MY Lee J Kissick HT Burger MC. Defining CD8 + T cells that provide the proliferative burst after PD-1 therapy. Nature (2016) 537:417–21. 10.1038/nature19330 27501248 PMC5297183

[25] Utzschneider DT Charmoy M Chennupati V Pousse L Ferreira DP Calderoncopete S. T cell factor 1-expressing memory-like CD8(+) T cells sustain the immune response to chronic viral infections. Immunity (2016) 45:415–27. 10.1016/j.immuni.2016.07.021 27533016

[26] Wu T Ji Y Moseman EA Xu HC Manglani M Kirby M. (2016). The TCF1-Bcl6 axis counteracts type I interferon to repress exhaustion and maintain T cell stemness. Sci Immunol. 1:eaai8593. 10.1126/sciimmunol.aai8593 28018990 PMC5179228

[27] McCausland M Crotty S. Quantitative PCR technique for detecting lymphocytic choriomeningitis virus *in vivo*. J Virol Methods (2008) 147:167–76. 10.1016/j.jviromet.2007.08.025 17920702 PMC2330273

[28] Wherry EJ Ha SJ Kaech SM Haining WN Sarkar S Kalia V. Molecular signature of CD8+ T cell exhaustion during chronic viral infection. Immunity (2007) 27:670–84. 10.1016/j.immuni.2007.09.006 17950003

[29] He R Hou S Liu C Zhang A Bai Q Han M. Follicular CXCR5-expressing CD8 + T cells curtail chronic viral infection. Nature (2016) 537:412–6. 10.1038/nature19317 27501245

[30] Pearce EL Mullen AC Martins GA Krawczyk CM Hutchins AS Zediak VP. Control of effector CD8+ T cell function by the transcription factor eomesodermin. Science (2003) 302:1041–3. 10.1126/science.1090148 14605368

[31] Ichii H Sakamoto A Kuroda Y Tokuhisa T. Bcl6 acts as an amplifier for the generation and proliferative capacity of central memory CD8+ T cells. J Immunol. (2004) 173:883–91. 10.4049/jimmunol.173.2.883 15240675

[32] Yoshida K Sakamoto A Yamashita K Arguni E Horigome S Arima M. Bcl6 controls granzyme B expression in effector CD8+ T cells. Eur J Immunol. (2006) 36:3146–56. 10.1002/eji.200636165 17125145

[33] Barber DL Wherry EJ Masopust D Zhu B Allison JP Sharpe AH. Restoring function in exhausted CD8 T cells during chronic viral infection. Nature (2006) 439:682–7. 10.1038/nature04444 16382236

[34] Meijer L Flajolet M Greengard P. Pharmacological inhibitors of glycogen synthase kinase 3. Trends Pharmacol Sci. (2004) 25:471–80. 10.1016/j.tips.2004.07.006 15559249

[35] Zhao D Yu S Zhou X Haring JS Held W Badovinac VP. Constitutive activation of Wnt signaling favors generation of memory CD8 T cells. J Immunol. (2010) 184:1191–9. 10.4049/jimmunol.0901199 20026746 PMC2809813

[36] Zhou X Xue H. Cutting edge: generation of memory precursors and functional memory CD8+ T cells depends on T cell factor-1 and lymphoid enhancer-binding factor-1. J Immunol. (2012) 189:2722–6. 10.4049/jimmunol.1201150 22875805 PMC3437003

[37] De Obaldia ME Bhandoola A. Transcriptional regulation of innate and adaptive lymphocyte lineages. Annu Rev Immunol. (2015) 33:607–42. 10.1146/annurev-immunol-032414-112032 25665079

[38] Jeannet G Boudousquie C Gardiol N Kang J Huelsken J Held W. Essential role of the Wnt pathway effector Tcf-1 for the establishment of functional CD8 T cell memory. Proc Natl Acad Sci USA. (2010) 107:9777–82. 10.1073/pnas.0914127107 20457902 PMC2906901

[39] Shin H Blackburn SD Intlekofer AM Kao C Angelosanto JM Reiner SL. A role for the transcriptional repressor blimp-1 in CD8+ T cell exhaustion during chronic viral infection. Immunity (2009) 31:309–20. 10.1016/j.immuni.2009.06.019 19664943 PMC2747257

[40] Masson F Minnich M Olshansky M Bilic I Mount AM Kallies A. Id2-mediated inhibition of E2A represses memory CD8+ T cell differentiation. J Immunol. (2013) 190:4585–94. 10.4049/jimmunol.1300099 23536629 PMC3631715

[41] Yang CY Best JA Knell J Yang E Sheridan AD Jesionek AK. The transcriptional regulators Id2 and Id3 control the formation of distinct memory CD8+ T cell subsets. Nat Immunol. (2011) 12:1221–9. 10.1038/ni.2158 22057289 PMC3872000

