# Supplementary material for: The Transcription Factor TCF1 Preserves the Effector Function of Exhausted CD8 T Cells During Chronic Viral Infection
Source: Front Immunol. 2019 Feb 12;10:169. doi: 10.3389/fimmu.2019.00169 (PMC6381939; doi:10.3389/fimmu.2019.00169)
Supplement: Supplementary file 1 [file Data_Sheet_1.docx]

Supplementary Material

The transcription factor TCF1 preserves the effector function of exhausted CD8 T cells during chronic viral infection

Yifei Wang^1#^, Jianjun Hu^2#^, Yiding Li^2^, Minglu Xiao, Haoqiang Wang^2^, Qin Tian, Zhirong Li^2^, Jianfang Tang^2^, Li Hu^2^, Yan Tan, Xinyuan Zhou^2^, Ran He^3^, Lilin Ye^2^, Yuzhang Wu^2^, Zhinan Yin^1,4*^, Qizhao Huang^5^*, Lifan Xu^2*^

*** Correspondence:** Zhinan Yin, Qizhao Huang, Lifan Xu: [zhinan.yin@yale.edu](mailto:zhinan.yin@yale.edu); [huangqizhao1988@163.com](mailto:huangqizhao1988@163.com); [xlftofu@sina.com](mailto:xlftofu@sina.com)

# Supplementary Figures and Tables

## Supplementary Figures


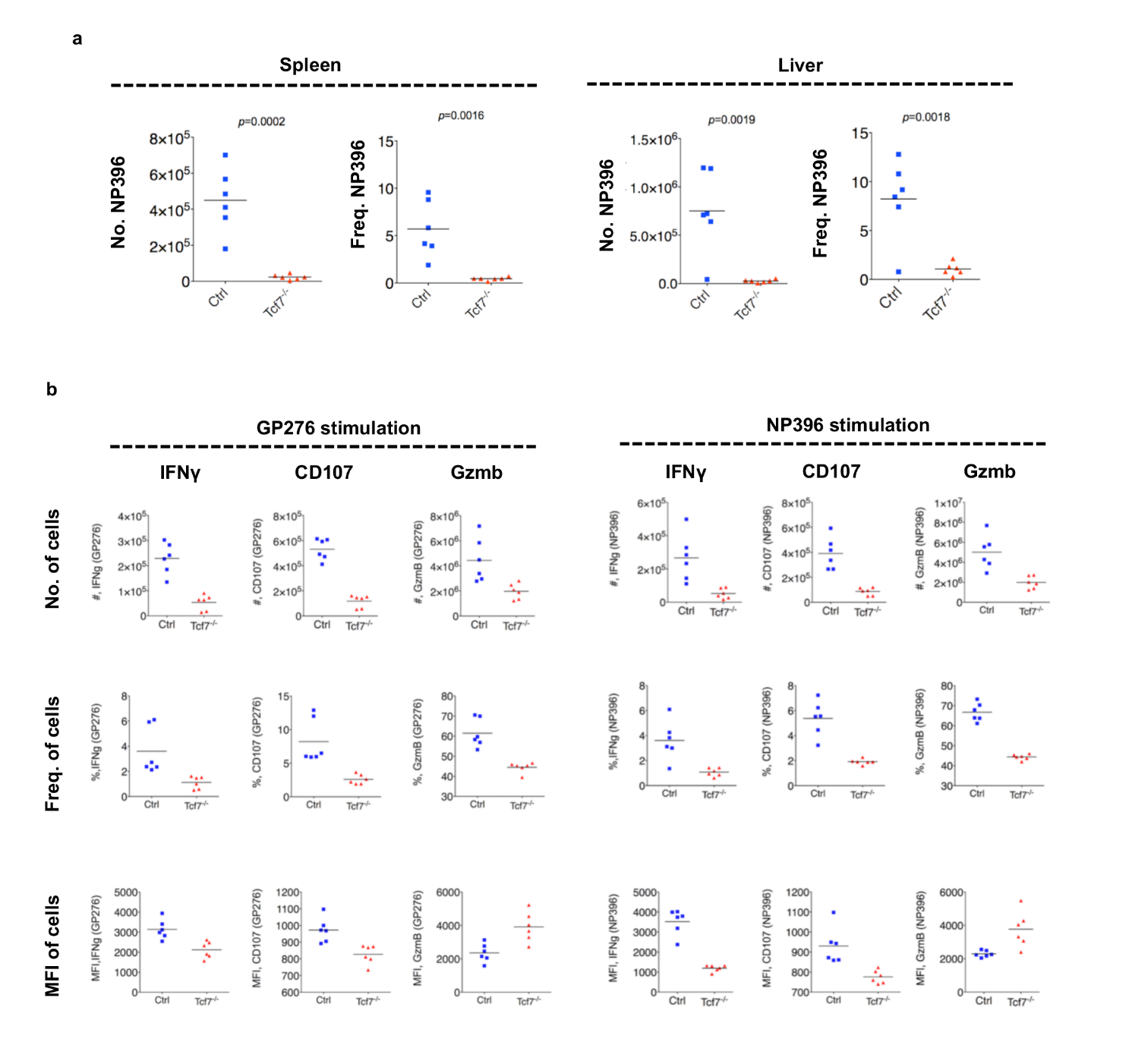


**Supplementary Figure 1. TCF1 deficiency exacerbates CD8 T cell exhaustion**

**(a)** Summary of number and percentage of NP396-tetramer positive CD8 T cells in the spleen or liver in either WT (Ctrl) or *Tcf7*^fl/fl^-CD4Cre (Tcf7^-/-^) mice. **(b)** Summary of cellular number and frequency of IFNγ-, CD107-, or Gzmb-positive CD8 T cells in the spleen of either WT (Ctrl) mice or *Tcf7*^fl/fl^-CD4Cre mice (Tcf7^-/-^) at day 8 post LCMV Cl13 infection after stimulation with LCMV peptide GP276 or NP396 *in vitro* for 5 hours. MFI of Gzmb, CD107, or IFNγ was calculated in those positive cell population. The *p* value was calculated by an unpaired t-test. Data are representative of three independent experiments with at least four or five mice per group.


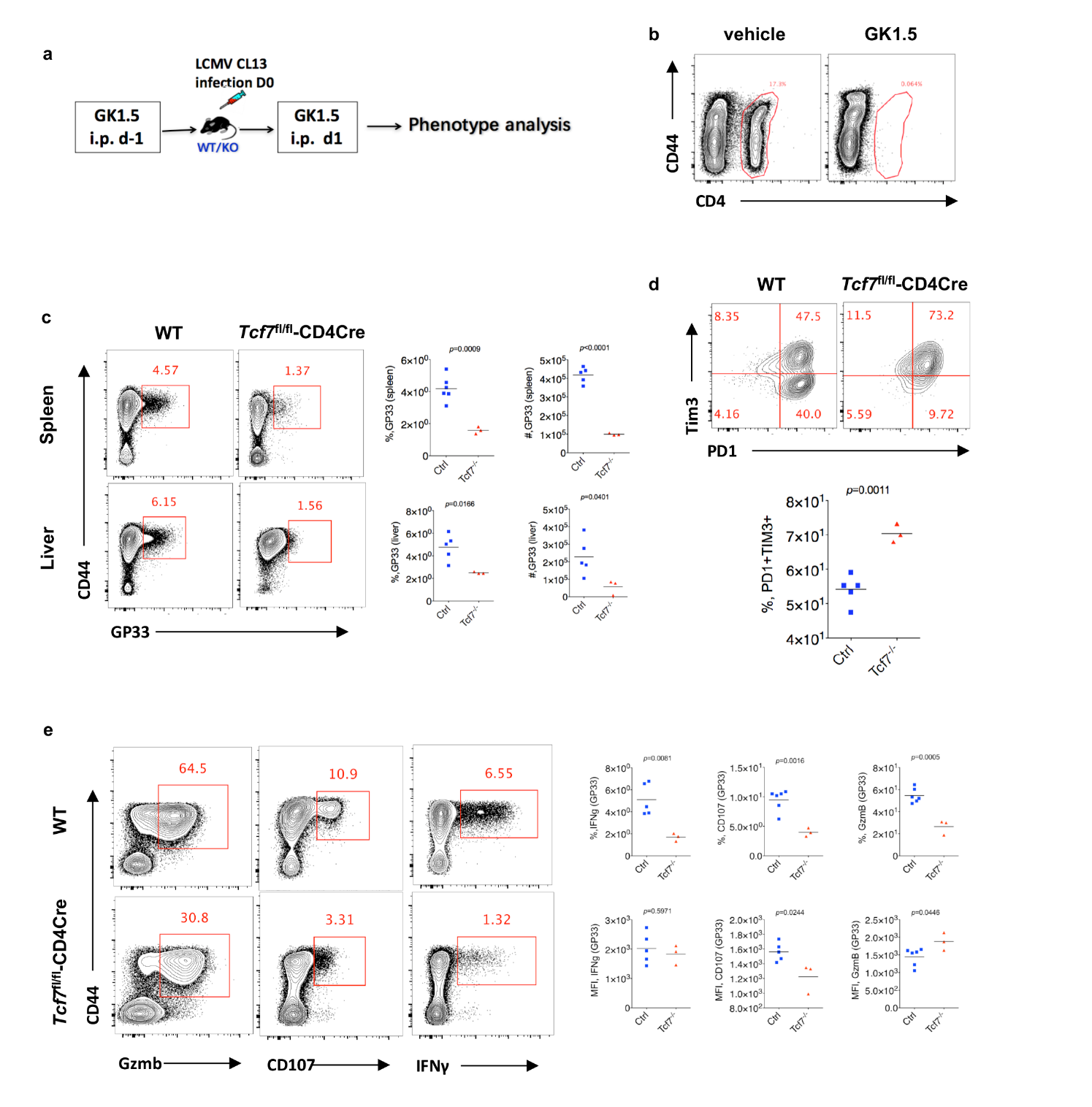


**Supplementary Figure 2. TCF1 regulates CD8 T cell responses independent on CD4 T cell**

**(a)** Experimental setup of depletion of CD4 T cells in WT (Ctrl) mice or *Tcf7*^fl/fl^-CD4Cre mice (KO, Tcf7^-/-^) mice via intraperitoneal injection with CD4 antibody (GK1.5). **(b)** Flow cytometry of CD4 T cells in mice at 2 days after injection with either GK1.5 or vehicle. **(c)** Flow cytometry of CD8 T cells in the spleen and liver in GK1.5 injected WT (Ctrl) mice or *Tcf7*^fl/fl^-CD4Cre mice (Tcf7^-/-^) at day 8 after LCMV Cl13 infection. Frequency and number of GP33-tetramer positive CD8 T cells are indicated. **(d)** Frequency of PD1^+^Tim3^+^ cells in GP33-tetramer CD8 T cells. **(e)** Flow cytometry of CD8 T cells in the spleen of mice as in C after stimulation with LCMV peptide GP33 *in vitro* for 5 hours. Frequency of Gzmb-, CD107- or IFNγ-positive cells in viral specific CD8 T cells and MFI of Gzmb, CD107, or IFNγ was calculated in those positive cell population. The *p* value was calculated by an unpaired t-test. Data are representative of three independent experiments with at least three or four mice per group.

**Supplementary Figure 3. TCF1 intrinsically regulates CD8 T cell responses at both early and advanced stages**

**(a)** Flow cytometric analysis of expression of CD107, IFNγ, or TNFα in viral specific WT and Tamoxifen-induced Tcf7-knock out CD8 T cells in the spleen of mice as in Figure 4A and 4E. **(b)** Experimental setup of generating bone marrow chimera mice via reconstituting irradiated WT recipient mice (CD45.1) with a mixture of congenitally marked bone marrow cells from *Tcf7*^fl/fl^-ERT2Cre donor mice (CD45.2, 30%) and WT donor mice (CD45.1, 70%). Chimera mice were treated with tamoxifen at day -4~0 before LCMV Cl13 infection (strategy II) or at day 10~13 post LCMV Cl13 infection (strategy I). **(c)** Frequency of GP33-tetramer positive CD8 T cells in spleen of mice as in b at day 8 post LCMV Cl13 infection. **(d)** Flow cytometric analysis of expression of CD107, IFNγ, or TNFα in viral specific WT and Tcf7^-/-^ CD8 T cells in the spleen of mice as in b after stimulation with LCMV peptide GP33 *in vitro* for 5 hours. The *p* value was calculated by a paired t-test. Data are representative of three independent experiments with at least three or four mice per group.

**Supplementary Figure 4. TCF1 deficiency regulates the transcriptional programs of exhausted CD8 T cell**

**(a)** Quantitative RT-PCR of the selected genes expressed in exhausted WT and Tcf7^-/-^ CD8 T cells from mice infected LCMV Cl13 stain, value of fold change is normalized to their expression in WT group. **(b)** Flow cytometric analysis of expression of selected genes in CD8 T cells in the spleen of bone marrow chimera mice as Figure 3A.

**Supplementary Figure 5. BIO-treatment upregulates the production of cytokines in viral specific CD8 T cells**

**(a)** Flow cytometric analysis of expression of CD107 and IFNγ in wild-type viral specific CD8 T cells in the spleen of mice treated with or without BIO as in Figure 6E after stimulation with LCMV peptide GP33 *in vitro* for 5 hours.

## Supplementary tables

**Supplementary table 1. Antibodies and reagents used in flow cytometry**

| **Antibody target/Reagent** | **Clone** | **Provider** |
| --- | --- | --- |
| CD4 | RM4-5 | Biolegend |
| CD8 | 53-6.7 | BD Biosciences |
| CD44 | IM7 | eBioscience |
| CD45.1 | A20 | Biolegend |
| CD45.2 | 104 | Biolegend |
| TCF-1 | C46C7 | Cell Signalling Technology |
| Tim3 | 215008 | R&D Systems |
| PD-1 | RMP1-30 | eBioscience |
| 2B4 | m2B4(B6)458.1 | Biolegend |
| B220 | RA3-6B2 | eBioscience |
| CXCR5 | 2G8 | BD Biosciences |
| Biotin Goat Anti-Rat IgG | 112-065-143 | Jackson Immunoresearch |
| Streptavidin | 25-4317-82 | eBioscience |
| Live/Dead Kit | L10119 | Life Technologies |
| Fc-blocker | 2.4G2 | BD Biosciences |
| Caspase 3 | C92-605 | BD Biosciences |
| Bcl-2 | 7/Bcl-2 | BD Biosciences |
| Annexin V Kit | 88-8102 | eBioscience |
| BrdU | 3D4 | BD Biosciences |
| Ki-67 | B56 | BD Biosciences |
| Granzyme B | GB11 | Life technologies |
| IFN γ | XMG1.2 | BD Biosciences |
| TNF α | MP6-XT22 | BD Biosciences |
| CD107a | 1D48 | BD Biosciences |
| CD107b | ABL-93 | BD Biosciences |
| Bcl-6 | K112-91 | BD Biosciences |
| EZH2 | 11/EZH2 | BD Biosciences |
| Eomes | Dan11mag | eBiosciences |
| Blimp-1 | 3H2-E8 | Novus Biologicals |

**Supplementary table 2. Primers used in quantitative PCR**

| **Gene symbol** | **Forward primer** | **Reverse primer** |
| --- | --- | --- |
| ***Cd107*** | cagcactctttgaggtgaaaaac | acgatctgagaaccattcgca |
| ***Gzmb*** | ccactctcgaccctacatgg | ggcccccaaagtgacatttatt |
| ***Perforin*** | agcacaagttcgtgccagg | gcgtctctcattagggagttttt |
| ***Ifgr*** | atgaacgctacacactgcatc | ccatccttttgccagttcctc |
| ***Tnfa*** | cctgtagcccacgtcgtag | ggagtagacaaggtacaaccc |
| ***Foxo1*** | cccaggccggagtttaacc | gttgctcataaagtcggtgct |
| ***Irf4*** | tccgacagtggttgatcgac | cctcacgattgtagtcctgctt |
| ***Pbx3*** | tggcagaaggggtttcagg | gggtcaatttggctctgtaatct |
| ***Ezh2*** | agcacaagtcatcccgttaaag | aattctgttgtaagggcgacc |
| ***Zeb2*** | attgcacatcagactttgaggaa | ataatggccgtgtcgcttcg |
| ***Nfatc1*** | gacccggagttcgacttcg | gacactaggggacacataactg |
| ***Eomes*** | gcaataagatgtacgttcaccca | gcagagactgcaacactatcat |
| ***Tcf7*** | caatctgctcatgccctacc | cttgcttctggctgatgtcc |
| ***Batf*** | ctccaggcttgatcgtctttt | cgggtatcccaatttgtactcc |
| ***Cxcr5*** | tggccttctacagtaacagca | gcatgaataccgccttaaaggac |
| ***Ccr5*** | atggattttcaagggtcagttcc | ctgagccgcaatttgtttcac |
| ***Ccr6*** | gctccagaacactgacgca | ctgtaccgtggctcacaga |
| ***Ccr7*** | gcccagatggtttttgggttc | gcaaggtacggatgataatgagg |
| ***Cxcr3*** | tctccctacgattatggggaaaa | ggttctgtcaaagttcaggct |
